# Effectiveness of First-Line Therapy with Old and Novel Antibiotics in Ventilator-Associated Pneumonia Caused by Carbapenem-Resistant *Acinetobacter baumannii*: A Real Life, Prospective, Observational, Single-Center Study

**DOI:** 10.3390/antibiotics12061048

**Published:** 2023-06-14

**Authors:** Lidia Dalfino, Monica Stufano, Davide Fiore Bavaro, Lucia Diella, Alessandra Belati, Stefania Stolfa, Federica Romanelli, Luigi Ronga, Rosa Di Mussi, Francesco Murgolo, Daniela Loconsole, Maria Chironna, Adriana Mosca, Maria Teresa Montagna, Annalisa Saracino, Salvatore Grasso

**Affiliations:** 1 Intensive Care Unit II, Department of Precision Medicine, Ionic Area, University of Bari “A. Moro”, 70124 Bari, Italy; monistufa@gmail.com (M.S.); rosselladimussi@libero.it (R.D.M.); francesco.murgolo@uniba.it (F.M.); salvatore.grasso@uniba.it (S.G.); 2 Clinic of Infectious Diseases, Department of Biomedical Sciences and Human Oncology, University of Bari “A. Moro”, 70124 Bari, Italy; davidebavaro@gmail.com (D.F.B.); diella.lucia@gmail.com (L.D.); alessandra.belati@hotmail.it (A.B.); annalisa.saracino@uniba.it (A.S.); 3 Microbiology and Virology Unit, Department of Interdisciplinary Medicine, University of Bari “A. Moro”, 70124 Bari, Italy; stolfastefania@gmail.com (S.S.); federicarosaromanelli@gmail.com (F.R.); rongalu@yahoo.it (L.R.); adriana.mosca@uniba.it (A.M.); 4 Hygiene Section, Department of Interdisciplinary Medicine, University of Bari “A. Moro”, 70124 Bari, Italy; daniela.loconsole@uniba.it (D.L.); maria.chironna@uniba.it (M.C.); mariateresa.montagna@uniba.it (M.T.M.)

**Keywords:** ventilator-associated pneumonia, carbapenem-resistant *Acinetobacter baumannii*, cefiderocol, colistin, nosocomial pneumonia, critically ill patients

## Abstract

Evidence-based, standard antibiotic therapy for ventilator-associated pneumonia (VAP) caused by carbapenem-resistant *Acinetobacter baumannii* (CRAB) is a relevant unmet clinical need in the intensive care unit (ICU). We aimed to evaluate the effectiveness of first-line therapy with old and novel CRAB active antibiotics in monomicrobial VAP caused by CRAB. A prospective, observational study was performed in a mixed non-COVID-19 ICU. The primary outcome measure was clinical failure upon first-line targeted therapy. Features independently influencing failure occurrence were also investigated via Cox proportional multivariable analysis. To account for the imbalance in antibiotic treatment allocation, a propensity score analysis with an inverse probability treatment weighting approach was adopted. Of the 90 enrolled patients, 34 (38%) experienced clinical failure. Compared to patients who experienced a clinical resolution of VAP, those who had clinical failure were of an older age (median age 71 (IQR 64–78) vs. 62 (IQR 52–69) years), and showed greater burden of comorbidities (median Charlson comorbidity index 8 (IQR 6–8) vs. 4 (IQR 2–6)), higher frequency of immunodepression (44% vs. 21%), and greater clinical severity at VAP onset (median SOFA score 10 (IQR 9–11) vs. 9 (IQR 7–11)). Lower rates of use of fast molecular diagnostics for nosocomial pneumonia (8.8% vs. 30.3%) and of timely CRAB active therapy administration (65% vs. 89%), and higher rates of colistin-based targeted therapy (71% vs. 46%) were also observed in patients who failed first-line therapy. Overall, CRAB active iv regimens were colistin-based in 50 patients and cefiderocol-based in 40 patients, both always combined with inhaled colistin. According to the backbone agent of first-line regimens, clinical failure was lower in the cefiderocol group, compared to that in the colistin group (25% vs. 48%, respectively). In multivariable Cox regression analysis, the burden of comorbid conditions independently predicted clinical failure occurrence (Charlson index aHR = 1.21, 95% CI = 1.04–1.42, *p* = 0.01), while timely targeted antibiotic treatment (aHR = 0.40, 95% CI = 0.19–0.84, *p* = 0.01) and cefiderocol-based first-line regimens (aHR = 0.38, 95% CI = 0.17–0.85, *p* = 0.02) strongly reduced failure risk. In patients with VAP caused by CRAB, timely active therapy improves infection outcomes and cefiderocol holds promise as a first-line therapeutic option.

## 1. Introduction

Ventilator-associated pneumonia (VAP) frequently complicates critically ill patient care [1]. In the last decade, carbapenem-resistant *Acinetobacter baumannii* (CRAB) has been among the pathogens that most frequently cause VAP [2]. Infection prevention, and control disruption and antibiotic overuse during the COVID-19 pandemic has further increased CRAB hospital spreading recently [3]. Crude mortality by severe infections is relevant [1], reaching rates as high as 50%, mainly driven by patients’ underlying high-risk conditions, VAP severity, delayed CRAB directed treatment [4] and the lack of scientifically validated effective antibiotics [5]. Currently, based on in vitro synergy studies, combinations of limited old treatment options among sulbactam, colistin, tigecycline and aminoglycosides are suggested as the front line, although supportive robust data on their clinical effectiveness are scarce [6,7], making the appropriate treatment of severe infections challenging. Concerning newly introduced antibiotics, a conditional recommendation against the treatment of CRAB infections with cefiderocol, the only novel beta-lactam with potent in vitro activity against CRAB, is provided, pending evidence-based efficacy data [6]. In this contemporary clinical scenario of a high unmet clinical need, real-life observational experiences may provide valuable insights. A mortality benefit provided by cefiderocol-based compared to colistin-based antibiotic therapy recently emerged in CRAB infections, including VAP, from clinical studies [8,9,10]. Investigating outcome measures reflecting antibiotic effectiveness more directly than mortality in the inherently frail patient population vulnerable to *Acinetobacter baumannii* represents a step forward. 

We aimed to investigate clinical failure upon first-line in vitro active antibiotics administered in real-life conditions to patients with monomicrobial VAP caused by CRAB and that features independently influencing failure occurrence.

## 2. Materials and Methods

### 2.1. Study Design, Setting and Participants

A prospective, observational, single-center study was performed in a 16-bed mixed non-COVID-19 intensive care unit (ICU) of the university tertiary care hospital “Policlinico” of Bari, Italy. All consecutive adult patients who developed VAP due to CRAB from 11 March 2021 to 31 December 2022 were eligible for the study. The exclusion criteria were as follows: age of <18 years, polymicrobial VAP, documented in vitro resistance to first-line antibiotic agents, and duration of first-line therapy of less than 72 h. Each patient contributed to one VAP case.

### 2.2. Data Collection

Demographics and clinical data on ICU admission were retrieved via the medical records of patients and included age, gender, typology of admission (medical or surgical), burden of comorbid conditions measured by the Charlson comorbidity index (CCI), immunodepression, and severity of critical illness measured by the Acute Physiologic Assessment and Chronic Health Evaluation (APACHE) II score. Patients were monitored daily and the following data were recorded: (1) time of onset of VAP from ICU admission; (2) daily clinical severity, measured via the worst value of the ratio of arterial O_2_ tension to inspired O_2_ fraction (PaO_2_/FiO_2_), the criteria of uncomplicated infection, sepsis and septic shock, and the Sequential Organ Failure Assessment (SOFA) score; (3) timeliness of in vitro active antimicrobial therapy and administered regimens (drug, daily dose and fractioning, and duration of therapy); (4) previous colonization by CRAB on endotracheal aspirate (ETA) surveillance cultures; (5) findings of fast molecular diagnostics for nosocomial pneumonia; (6) the antimicrobial susceptibility profile of the isolated CRAB and its reporting time; (7) follow-up ETA or bronchoalveolar lavage (BAL) cultures; (8) the occurrence of secondary bacteraemia, acute kidney injury (AKI) or augmented renal clearance (ARC), need for renal replacement therapy (RRT) and/or veno-venous extracorporeal membrane oxygenation (vv-ECMO) at the onset or during the VAP course; (9) clinical failure, microbiological failure, 14-day mortality, 28-day mortality, and ICU length of stay (LOS).

Data were anonymously recorded in an electronic dataset. Patients were followed up until 28 days from infection onset or ICU death/discharge, whichever came first.

### 2.3. Definitions

The term CRAB refers to carbapenem-resistant *A. baumannii* and other species within the *A. baumannii* and *calcoaceticus* complexes. Carbapenem resistance was defined according to EUCAST criteria [11].

VAP diagnosis was given to patients receiving invasive mechanical ventilation for at least 48 h, presenting new or progressive and persistent infiltrate, consolidation, or cavitation upon chest imaging, associated with at least two of the following criteria: a body temperature of >38 °C or <36 °C, a white blood cell count of ≥12.000 cells/mm^3^ or ≤4.000 cells/mm^3^ and purulent tracheal aspirate. Worsening oxygenation after a period of stability or improvement, requiring an increase in daily minimum FiO_2_ of ≥0.20 and/or in daily minimum positive end-expiratory pressure (PEEP) of ≥3 cmH_2_O, sustained for 2 or more days, was also required for diagnosis. Microbiological confirmation required CRAB isolation upon ETA or BAL culture, meeting quantitative thresholds of pathogen growth of >10^5^ CFU/mL and >10^4^ CFU/mL, respectively [12,13].

Bacteraemic VAP was defined as isolation of CRAB in at least one blood culture, in the absence of other known sources of bacteraemia. Polymicrobial VAP was defined as isolation of *A. baumannii* plus any other bacterial or fungal pathogen upon ETA or BAL via a multiplex real time-polymerase chain reaction (RT-PCR) respiratory panel and/or culture. Infection severity was stratified into uncomplicated infection, sepsis, and septic shock, according to Sepsis-3 consensus definitions [14].

Timely first-line targeted therapy was defined as the administration of at least one CRAB in vitro active agent within 24 h from VAP onset. 

Clinical failure was defined as (1) the need to switch to second-line antibiotic therapy due to a lack of clinical response, defined by more than one of the following criteria: persistence of fever or hypothermia, no improvement or worsening of PaO_2_/FiO_2_, persistence of purulent respiratory secretions, increase in pulmonary infiltrates upon a chest radiograph of >50%, clinical worsening along the continuum of uncomplicated infection, sepsis and septic shock, and multiple-organ dysfunction syndrome occurrence, defined as at least 2 organ system failures not present on day 1; or (2) recurrence of VAP caused by CRAB up to 7 days from the end of active antimicrobial therapy.

Clinical resolution was defined as recovery from VAP, as defined above, at the end of first-line therapy.

Microbiological failure was defined as the persistence of CRAB for ≥7 days from starting first-line active therapy upon ETA or BAL follow-up cultures.

Acute kidney injury (AKI) was defined according to 2012 KDIGO criteria [15]. De novo AKI was defined as AKI occurring for >48 h from the start of therapy. Augmented renal clearance was defined as a creatinine clearance of ≥130 mL/min/1.73 m^2^ [16].

Immunodepression included neutropenia (neutrophil count, <500 cells/mm^3^), solid cancer upon chemotherapy, solid organ transplantation, hematopoietic stem cell transplantation, corticosteroid therapy at a dosage of >16 mg/day of prednisone for at least 15 days or other chronic immunosuppressive therapies, and uncontrolled HIV infection (CD4 cell count of <200 cells/mm^3^).

### 2.4. Procedures and Antimicrobial Treatment

As for institutional policy, an infection control program was active and included twice-weekly surveillance cultures on ETA specimens during ICU stays. At the onset of VAP, BAL and blood cultures were performed in all patients and a multiplex RT-PCR respiratory panel was conducted on BAL samples in patients with immunodepression, severe hypoxemia (PaO_2_/FiO_2_ < 100) or septic shock.

According to current guidelines [17,18], timely hemodynamic monitoring via volumetric index measurement and transpulmonary thermodilution was started in patients with sepsis-induced hypoperfusion or septic shock.

The choice of antibiotic therapy was at the joint discretion of the treating physician and an expert in the field of ICU infections. At VAP onset, antibiotic therapy was based on individual patient assessment (risk factors for multidrug-resistant organisms, carriage status, rapid molecular tests findings, and clinical severity), according to a treatment algorithm adapted to the local epidemiology and regularly updated by the ICU antimicrobial stewardship (AMS) team, encompassing two intensivists, three ID consultants and three microbiologists. Prescribed regimens were then endorsed by the AMS team in the daily morning rounds.

Intravenous (iv) antibiotics were administered with the daily doses and fractioning recommended for critically ill patients including tigecycline, with a first dose of 200 mg, followed by 100 mg every 12 h infused over 60 min; ampicillin/sulbactam, with a loading dose (LD) of 6 g/3 g followed by a 4 h infusion of 6 g/3 g every 8 h; meropenem, with a 2 g LD followed by a 6 h infusion of 2 g every 8 h; fosfomycin, with a 6 g LD followed by a continuous infusion of 16–24 g daily [7,19]. Colistin was administered as colistimethate sodium (CMS) and dosed according to current international guidelines [20]. Patients treated with colistin received 1 g of ascorbic acid 30 min before iv colistin administration, to prevent colistin-induced nephrotoxicity [21]. Cefiderocol was administered as a LD of 2 g followed by a 8 h infusion of 2 g every 8 h in patients with normal renal function and by a 6 h infusion of 2 g every 6 h in patients with ARC [22,23]. For all administered antibiotics, maintenance dose adjustments were performed in patients with renal impairment, according to the manufacturer’ recommendations. In patients without a history of chronic kidney disease who presented AKI at VAP onset, beta-lactam standard doses were administered and titration to renal function was performed after 48 h from the start of therapy only if persistent AKI was documented [24]. 

Regardless of the type of iv regimens, all patients received inhaled colistin. A fixed dose of 2 million international units of CMS diluted in 5 mL of normal saline was administered every 8 h with a vibrating mesh nebulizer placed 10 cm before the Y-piece in a ventilator circuit. During nebulization, the heated humidifier was switched off, to reduce aerosol inertial impaction in the ventilator circuit and maximize delivery to the patient. Mechanical ventilation was set to controlled mode, with a tidal volume of 6 mL/kg of predicted body weight. The mechanical filter placed on the expiratory limb of the circuit was changed at the end of each nebulization, to avoid clogging [25].

### 2.5. Microbiological Identification and Susceptibility Testing

Fast molecular diagnostics for nosocomial pneumonia was performed on BAL samples with a commercial multiplex qPCR system (Biofire Filmarray^®^ Pneumonia plus panel, biòMerieux, Marcy l’Étoile, France).

Isolate identification and antimicrobial susceptibility testing were performed via matrix-assisted laser desorption ionization (MALDI) time-of-flight (ToF) mass spectrometry (MALDI MS^®^, biòMerieux, Lyon, France) and the Vitek^®^2 automated system (biòMerieux, Lyon, France), respectively. Colistin susceptibility testing was conducted via broth microdilution. Minimum inhibitory concentration (MIC) values were interpreted according to clinical breakpoints established by the European Committee on Antimicrobial Susceptibility Testing (EUCAST) [26]. Cefiderocol susceptibility testing was performed through the disk diffusion method using Cefiderocol 30 µg discs (Liofilchem^®^, Roseto degli Abruzzi (TE), Italy) in standard Mueller–Hinton agar plates. Inhibition zone diameters of ≥17 mm for cefiderocol corresponded to MIC values below the PK/PD breakpoint of sensitivity, ≤ 2 mg/L [27]. Fosfomycin was not tested due to the intrinsic resistance of *A. baumannii*.

### 2.6. Study Outcomes

The primary outcome measure of the study was clinical failure upon first-line CRAB active antimicrobial therapy. Independent predictors of clinical failure were also investigated. Secondary outcome measures were clinical and microbiological failure rates, and the 14-day and 28-day mortality of patients stratified by first-line regimens. For the assessment of outcomes, day 1 was defined as the day of VAP onset.

### 2.7. Statistical Analysis

According to their distribution evaluated by the Kolmogorov–Smirnov test, continuous data are reported as the median and interquartile range (IQR) and compared using the Mann–Whitney U test. Categorical data are reported as absolute values and percentages and compared using the χ^2^ test or Fisher’s exact test, as appropriate. To investigate independent predictors of clinical failure, a Cox proportional hazard model was performed. Variables with a significant endpoint (*p* value < 0.1) upon univariable Cox regression analysis were included in a multivariable stepwise analysis, to control for potential confounders. Crude (HR) and adjusted hazard ratios (aHR) and 95% confidence intervals (95% CI) were reported. 

Since cefiderocol-based first-line regimens turned out to independently reduce clinical failure risk compared to colistin-based regimens, an inverse probability treatment weighting (IPTW) analysis was performed, to deal with the potential treatment selection bias in the non-randomized assignment of included patients to cefiderocol or colistin groups. A multivariable logistic regression model was produced to obtain, for each patient, the propensity score (PS) of receiving cefiderocol-based therapy. The covariates included in the model were CCI, immunodepression, SOFA score, septic shock, bacteraemia, ARC, need for RRT, and timely antimicrobial treatment, which were available prior to treatment assignment and potentially influenced both the treatment decision and the outcome. To avoid collinearity with the CCI, age was not included in the model. The contribution of each patient was weighted by the inverse probability of receiving cefiderocol-based treatment for patients in cefiderocol group, whereas it was weighted by the inverse of 1 minus the PS for patients in the colistin group. Weighting generated a pseudo-population in which the influence of patients receiving a treatment they would not be expected to receive was increased, producing a mathematical representation of “rare” patients in each treatment group [28]. Standardized differences (SD) were used to quantitatively compare the balance in measured baseline covariates between the cefiderocol group and colistin group in both the unweighted and weighted sample. SD values below 10% suggested a negligible imbalance between groups, indicating no further adjustment requirement in outcome analyses [29]. Cox proportional regression analysis of the IPTW population was then performed and an IPTW aHR (95% CI) of cefiderocol based treatment on clinical failure was reported. 

Statistical analysis was performed using STATA v17 (StataCorp, College Station, TX, USA), and statistical significance was set at a *p*-value of < 0.05.

## 3. Results

### 3.1. Clinical Characteristics of Patients Stratified by Primary Outcome

Out of the 981 patients consecutively admitted to our ICU from March 2021 to December 2022, 129 patients developed VAP caused by CRAB. In total, 39 patients were excluded from analysis, 31 patients being excluded due to polymicrobial VAP, and 8 patients being excluded due to a treatment duration of less than 72 h, because of early death or transfer to other ICUs. Therefore, 90 patients were enrolled. 

Clinical failure upon first-line targeted therapy was observed in 34 (38%) patients. All events were due to a lack of clinical response at a median of 6 days (IQR 4–8) from starting therapy. No VAP recurrence was observed. Compared to those in the clinical resolution group, those in the clinical failure group were of an older age, had more frequent immunodepression, and presented a higher CCI and higher SOFA score at VAP onset (Table 1). Moreover, a less frequent etiological diagnosis made via rapid molecular diagnostics and timely first-line targeted therapy was observed in patients who failed first-line therapy, compared to those who had resolved VAP. The reporting time of the in vitro susceptibility profile of CRAB isolates did not differ between groups (3 (IQR 3-4) days in both groups). The clinical failure group was more frequently treated with colistin-based regimens. Specifically, a higher rate of colistin-tigecycline administration and a lower rate of cefiderocol–fosfomycin use were observed in these patients (Table 1). Finally, compared to those who underwent clinical resolution, patients who failed first-line therapy showed higher 14 d and 28 d mortality and a shorter length of ICU stay (Table 1).

### 3.2. Characteristics and Outcomes of Patients Stratified by First-Line Antibiotic Regimen

First-line targeted regimens administered during the study period were colistin-based in 50 (56%) patients (colistin group) and cefiderocol-based in 40 (44%) patients (cefiderocol group). Features of patients stratified by first-line antimicrobial regimens are reported in Table 2. No differences were found between cefiderocol and colistin groups regarding demographic and clinical characteristics on ICU admission, except for a higher occurrence of male gender and a higher clinical severity of patients who received cefiderocol-based regimens (Table 2). Regarding VAP characteristics, the study groups were also comparable in time of onset from ICU admission, PaO_2_/FiO_2_, SOFA score, sepsis, and septic shock at VAP onset, secondary bacteraemia, and need for extracorporeal supports (Table 2). Frequency of known CRAB carrier rates, etiological diagnosis made by fast molecular tests and timely targeted antimicrobial therapy was also not different between groups (Table 2). In cefiderocol group, 19 (47.5%) patients were treated with iv monotherapy, and 21 (52.5%) received combination iv therapy with fosfomycin as partner drug. In colistin-group, all patients received combination iv therapy, with tigecycline in 27 (54%) cases, ampicillin/sulbactam in 15 (30%) and meropenem in 8 (16%) cases. All patients in both groups received inhaled colistin.

According to EUCAST criteria [26,27], the susceptibility of CRAB isolates to colistin and cefiderocol was 100%. Colistin MIC values ranged from 0.5 to 1 mg/L. The cefiderocol zone diameter ranged from 19 to 22 mm in disk diffusion tests. 

**Figure 1:**
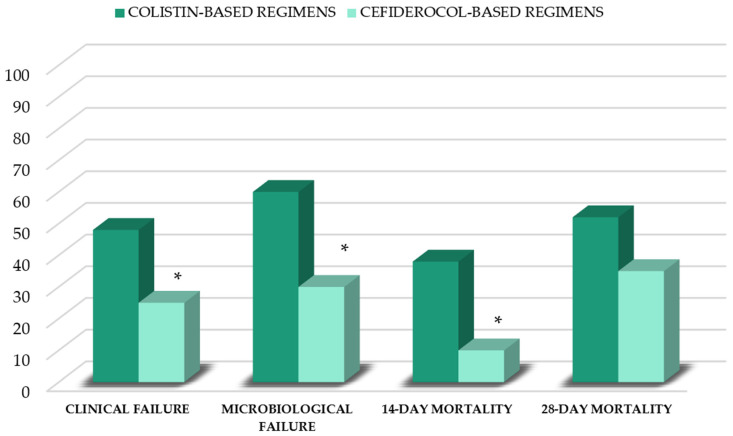
Outcomes of patients stratified by first-line therapy. * *p* < 0.05 vs. colistin group.

In patients who had resolved VAP, the median duration of antibiotic therapy did not differ between cefiderocol and colistin groups (Table 2). Compared to patients who received colistin-based regimens, patients who received cefiderocol-based regimens showed a lower clinical failure rate (25% vs. 48%; *p* = 0.02). Microbiological failure was not evaluable in 15 patients (10 in the colistin group and 5 in the cefiderocol group), due to a switch to second-line agents <7 days from the start of therapy. In the remaining 75 patients, microbiological failure was lower in the cefiderocol group (30% vs. 60%; *p* = 0.003). Finally, a lower 14 d mortality rate in the cefiderocol group, compared to that of the colistin group (10% vs. 38%; *p* = 0.03), was observed (Figure 1). There were no significant differences regarding de novo AKI occurrence (42% vs. 47%), length of ICU stay (16 (IQR, 12–21) days vs. 15 (12–17) days) and 28-day mortality rates between groups (35% vs. 52%) (Figure 1).

### 3.3. Independent Predictors of Clinical Failure

In the multivariable Cox regression analysis, the Charlson comorbidity index independently predicted clinical failure, while timely targeted antibiotic therapy and cefiderocol-based first-line targeted therapy turned out to independently reduce failure risk (Table 3).

These findings were confirmed through the IPTW analysis, with an IPTW aHR of 0.37 (95% CI 0.18–0.76, *p* = 0.007) for cefiderocol-based regimens. The IPTW-adjusted multivariable Cox model for predictors of clinical failure and standardized differences before and after IPT weighting are reported in the Supplementary Materials (Table S1 and Table S2, respectively).

## 4. Discussion

In a large cohort of patients with monomicrobial VAP caused by CRAB, clinical failure upon first-line targeted therapy involved nearly 40% of patients and was associated with 14-day and 28-day ICU mortality rates as high as 41% and 71%, respectively. These figures were 0% and 21% when clinical resolution of VAP was observed. Clinical failure rates significantly differed depending upon the backbone agent of targeted regimens administered in the front line, at 48% in patients who received colistin-based and 25% in those treated with cefiderocol-based regimens. Underlying comorbid conditions independently predicted clinical failure occurrence, whereas timely targeted therapy and cefiderocol-based first-line regimens strongly reduced failure risk.

VAP caused by CRAB frequently affects patients with a poor physiological background. Our study confirms the role of underlying host conditions in poor infection outcomes, as previously reported [4]. Noteworthily, however, regardless of the patient comorbidity burden, a protective role against clinical failure for modifiable features of the front-line approach to VAP caused by CRAB emerged.

Indeed, nearly 90% of patients who experienced infection resolution received CRAB active agents within 24 h from VAP onset and timely targeted therapy turned out to independently reduce the clinical failure risk by 60%. This finding is not surprising and is consistent with previous studies [4]. In our study cohort, 58% patients were colonized by CRAB and, accordingly, they received local susceptibility pattern-guided empirical treatment decisions, as recommended [7,12]. Remarkably, however, the relevant rates of timely active therapy, which prompted VAP resolution, were reached by the significant contribution of fast molecular diagnostics performed in high-risk patients. This finding substantiates the value of RT multiplex respiratory panels, which includes, in addition to withholding antibiotics, helping clinicians to promptly direct therapy in patients with severe VAP [30].

Along with timeliness, the choice of cefiderocol in the front line strongly reduced clinical failure chances in our study cohort. A unanimous consensus on the optimal antibiotic treatment of VAP caused by CRAB is still lacking [6,7]. So far, colistin remains the most widely employed backbone agent of targeted regimens, despite concerns regarding its ability to safely achieve adequate lung exposure [31], renal toxicity [32] and sub-optimal clinical response rates [33]. Indeed, 46.5% clinical response rates and 51% infection-related mortality rates have been reported in patients with VAP caused by CRAB treated with colistin-based regimens [33]. By virtue of strong in vitro activity against CRAB [34], and a favorable intrapulmonary PK [35,36] and safety profile, cefiderocol has been increasingly employed in clinical practice for CRAB infections, including pneumonia [8,9,10]. Even though in the phase III CREDIBLE-CR study mortality rates in the subset of CRAB infections were found to be higher in the cefiderocol arm, compared to the arm with the best available therapy [37], favorable insights emerged from real-life observational studies involving ICU patients [8,9,10]. In a large cohort of COVID-19 ICU patients with bloodstream or lower respiratory tract CRAB superinfections, cefiderocol monotherapy, administered on a compassionate use basis, was associated with a non-significantly lower 28-day mortality risk compared to colistin-based regimens [8]. In the largest retrospective analysis reported to date of 124 COVID-19 ICU patients with bloodstream infections or VAP caused by CRAB, 30-day mortality was 55.8% in patients who received colistin and 34% in those treated with cefiderocol-containing regimens. Upon multivariable analysis, cefiderocol-based therapy independently reduced mortality risks. Although this outcome advantage was not confirmed in patients with VAP, they represented only 28% of the study sample, and in half of them VAP was polymicrobial [9]. Finally, in the most recently published retrospective analysis of 73 COVID-19 patients with monomicrobial bacteraemic VAP caused by CRAB, 30-day mortality was 98% in patients treated with colistin-containing regimens and 32% in those who received cefiderocol-based regimens. Of note is that the latter were independently associated with 30-day survival [10].

Our study corroborates these findings and complements them, enriching the body of knowledge from real-world care settings in different ways, first by focusing on VAP, thus investigating a homogenous cohort of ICU patients with a unique infection site from the antibiotics pharmacokinetic/pharmacodynamic (PK/PD) perspective [31]. Secondly, we aimed for an earlier primary outcome measure, thus addressing antibiotic effectiveness more directly than mortality. This is of importance in patients whose baseline mortality risk is already high, as critically ill patients vulnerable to *A. baumanni*. Third, we analyzed CRAB active agents administered in the front line, thus obviating the challenging interpretation of effectiveness in a rescue therapy setting. Fourth, we excluded COVID-19 patients, thus avoiding the confounding effect of SARS-CoV2 pneumonia on both the diagnosis and outcome of VAP [38]. Finally, we focused on monomicrobial VAP, hence overcoming the uncertainty related to the unclear pathogenetic role of *A. baumannii* in polymicrobial infections [39]. Therefore, for the first time, in the general ICU population with monomicrobial VAP caused by CRAB, a contemporary clinical scenario with a high unmet treatment need, cefiderocol administration as a first-choice backbone agent was found to reduce clinical failure risk by almost 60%, being more effective than iv colistin.

By interpreting this finding, our beta-lactam dosing strategy should not be disregarded. In patients with VAP, early adequate antibiotic therapy drives infection outcomes [40]. Pneumonia, as do other deep-seated infections, pose a high risk of under-exposure to standard dosing beta-lactam antibiotics [41], which may, in turn, translate to clinical failure [42]. In mechanically ventilated patients with severe pneumonia, at standard dosing and fractioning, cefiderocol exposure in epithelial lining fluid is comparable to that in other cephalosporins [36] and population PK modeling suggests a high probability of achieving PK-PD targets when MICs are < 2 mg/L [36]. However, cefiderocol efficacy targets for CRAB are considerably higher than those for other carbapenem-resistant pathogens. Notably, a suboptimal attainment of those targets accounted for most cases of microbiological failure with cefiderocol in a case series of VAP caused by XDR *A. baumannii* that was recently reported [43]. Beta-lactams are known to exhibit time-dependent killing and better target attainments and clinical outcomes have been related to prolonged or continuous compared to standard infusion [44,45]. As for our institutional protocol, beta-lactams, including cefiderocol, are administered via continuous infusion preceded by a loading dose [23]. Thus, our dosing approach may possibly have favored timely optimal cefiderocol lung exposure [43], contributing to the low rates of clinical failure observed in our cohort, as has been reported for the prolonged infusion of ceftazidime–avibactam in infections caused by carbapenem-resistant *Enterobacterales* [42].

Likewise, it may be that our practice of administering beta-lactams at standard daily doses in the first 48 h of therapy in patients with AKI and without a history of chronic kidney disease influenced the timely achievement of adequate cefiderocol lung exposure in the early treatment phase. Indeed, transient AKI occurs in up to 46% patients with pneumonia-related renal injury [46] and a lower effectiveness of some novel beta-lactams among patients with moderate acute renal impairment at the onset of infection, compared to that among those with normal renal function [42], has been associated with inappropriate antibiotic dose reduction in the setting of transient AKI [46]. Thus, a deferral of dose titration to renal function when transient AKI was a possibility, as previously suggested [44,46], might have further improved the likelihood of clinical resolution in our cefiderocol-treated patients.

According to current guidelines [6,7], in our study cohort, colistin was always administered in combination therapy, with high-dose tigecycline as the most frequent partner agent. Nearly 50% patients who failed first-line therapy received colistin–tigecycline regimens. Cefiderocol was administered in combination with fosfomycin in over half of the patients. This was the most frequently employed combination regimen in patients who showed a clinical resolution of VAP. Whilst our study does not allow any conclusion regarding colistin and cefiderocol partner agents, due to the limited sample size, these findings are consistent with previous concerns about the performance of tigecycline in VAP [47] on one hand, and support the recently reported favorable experience with fosfomycin as a cefiderocol partner drug in VAP caused by CRAB [10] on the other hand.

Inhaled colistin administration for VAP caused by CRAB is highly debated [12,20]. In our whole cohort, inhaled colistin was employed as an adjunctive therapy to reduce bacterial burden, independently of administered CRAB active iv regimens. Therefore, any inference could be made on its effectiveness from the present study.

Regarding colistin, renal safety is a major concern. Consistently with previous studies [21], in our study cohort, de novo AKI occurred in 47% of patients who received colistin. Interestingly, comparable AKI rates in patients treated with cefiderocol were observed. It should not be disregarded, however, that this study was not designed to investigate renal toxicity caused by CRAB active agents and, as such, did not control for confounders. In ICU patients, sepsis accounts for 40% of AKI cases [48] and over 80% of patients in our cohort had sepsis or septic shock. Moreover, co-administered nephrotoxins and renal protective measures routinely employed in our ICU [21,49] were not measured. Thus, although we tried to differentiate sepsis-induced AKI, i.e., AKI already present at the onset of VAP, from renal injury observed later during therapy, whether or not AKI was a consequence of exposure to nephrotoxic antibiotics per se cannot be clearly ascertained by our data.

We acknowledge that our study has some relevant limitations. First, owing to the single-center design and the relatively small sample size, results may not be generalizable. Second, cefiderocol was available at our center from August 2021. Therefore, from March to July 2021, colistin-based regimens have been administered. Although a propensity score analysis with the IPTW approach was adopted to deal with a potential treatment selection bias, this possibility exists. Third, we acknowledge that ampicillin–sulbactam is suggested as the first-line backbone agent for severe CRAB infections [6]. However, its use is discouraged in settings with high-level sulbactam resistance, as it is in our ICU/hospital. Finally, susceptibilities to cefiderocol and colistin were not systematically tested in patients who experienced clinical failure. In our opinion, this is a major limitation of this study, considering the treatment-emergent cefiderocol resistance recently reported [9].

Despite these limitations, several methodological features of this study strengthen its findings and, for the first time, clinical failure upon first-line therapy for VAP caused by CRAB with colistin-based or cefiderocol-based regimens outside the COVID-19 setting has been reported.

## 5. Conclusions

In real-life conditions, the timeliness of CRAB active antibiotic administration and cefiderocol use as a first-line choice substantially reduces clinical failure risk in severe VAP caused by CRAB. Further studies are needed to corroborate these findings and to investigate fosfomycin as a cefiderocol partner agent in VAP caused by CRAB. The beneficial role of rapid molecular diagnostics in improving timely appropriate targeted therapy in high-risk patients with severe VAP deserves further investigations too.

## Figures and Tables

**Table 1:** Characteristics and outcome of study patients according to clinical failure or resolution upon first-line CRAB active therapy.

	Clinical Resolution (*n* = 56)	Clinical Failure (*n* = 34)
**Age (years)**	62 (52–69)	71 (64–78) *
**Male sex**	38 (68)	16 (47)
**Surgical admission**	30 (54)	18 (53)
**Immunodepression**	12 (21)	15 (44) *
**Charlson comorbidity index**	4 (2–6)	8 (6–8) *
**Main comorbidities**		
Diabetes mellitus	8 (14)	17 (50) *
Cardiovascular disease	13 (23)	18 (53) *
Chronic respiratory disease	6 (11)	15 (44) *
Chronic kidney disease	4 (7)	6 (18)
Chronic liver disease	2 (4)	3 (9)
Solid cancer	6 (11)	5 (15)
Active hematologic malignancies	2 (4)	5 (15)
Solid organ transplantation	3 (5)	6 (18)
Obesity (BMI > 30 kg/m^2^)	5 (9)	4 (12)
**APACHE II score upon ICU admission**	22 (20–25)	23 (20–25)
**VAP onset from ICU admission (days)**	8 (6–11)	9 (7–11)
**SOFA score at VAP onset**	9 (7–11)	10 (9–11) *
**Oxygenation at VAP onset**		
PaO_2_ to FiO_2_ ratio >200	9 (16)	4 (12)
PaO_2_ to FiO_2_ ratio >100 and <200	41 (73)	26 (76)
PaO_2_ to FiO_2_ ratio <100	6 (11)	4 (12)
**Infection severity at VAP onset**		
Uncomplicated infection	13 (23)	2 (6) *
Sepsis	19 (34)	10 (29)
Septic shock	25 (45)	22 (65)
**Bacteraemic VAP**	15 (26.8)	14 (41.2)
**Augmented renal clearance**	10 (18)	5 (15)
**CRRT**	8 (14)	8 (24)
**vv-ECMO**	3 (5)	1 (3)
**Known respiratory CRAB colonization**	34 (61)	18 (53)
**Fast molecular diagnostics at VAP onset**	17 (30.3)	3 (8.8) *
**Timely (≤24 h) targeted therapy**	50 (89)	22 (65) *
**Cefiderocol-based regimens**	30 (54)	10 (29) *
Cefiderocol–inhaled colistin	10 (17.8)	9 (26.5)
Cefiderocol–fosfomycin–inhaled colistin	20 (35.7)	1 (3) *
**Colistin-based regimens**	26 (46)	24 (71) *
Colistin–tigecycline–inhaled colistin	11 (20)	16 (47) *
Colistin–ampicillin/sulbactam–inhaled colistin	8 (14)	7 (21)
Colistin–meropenem–inhaled colistin	7 (13)	1 (3)
**14-day mortality**	0 (0)	14 (41) *
**28-day mortality**	12 (21)	24 (71) *
**ICU length of stay (days)**	24 (21–28)	21 (17–25) *

Data are presented as No. (%) of included patients or as median (interquartile range), unless otherwise indicated. * *p* < 0.05 vs. clinical resolution group. APACHE: Acute Physiologic Assessment and Chronic Health Evaluation; BMI: Body Max Index; CRAB: carbapenem-resistant *Acinetobacter baumannii*; CRRT: Continuous Renal Replacement Therapy; SOFA: Sequential Organ Failure Assessment; VAP: Ventilator Associated Pneumonia; vv-ECMO: veno-venous Extra-Corporeal Membrane Oxygenation.

**Table 2:** Demographic and clinical characteristics of study patients according to first-line treatment regimens.

	Cefiderocol Group (*n* = 40)	Colistin Group (*n* = 50)
**Age (years)**	67 (59–72)	64 (55–76)
**Male gender**	35 (86)	19 (38) *
**Surgical admission**	20 (50)	28 (56)
**Immunosuppression**	13 (33)	14 (28)
**Charlson Comorbidity Index**	5 (2–6)	7 (2–8)
**Comorbidities**		
Diabetes mellitus	7 (18)	18 (36)
Cardiovascular disease	12 (30)	19 (50)
Chronic respiratory disease	6 (15)	15 (30)
Chronic kidney disease	3 (8)	7 (14)
Chronic liver disease	1 (3)	1 (2)
Solid cancer	5 (13)	6 (12)
Active hematologic malignancies	3 (8)	4 (8)
Solid organ transplantation	4 (10)	5 (10)
Obesity (BMI > 30 kg/m^2^)	4 (10)	5 (10)
**APACHE II score on ICU admission**	24 (22–29)	22 (20–24) *
**VAP onset from ICU admission (days)**	8 (6–12)	9 (7–10)
**SOFA score at VAP onset**	9 (8–12)	9 (8–11)
**Oxygenation at VAP onset**		
PaO_2_ to FiO_2_ ratio >200	6 (15)	7 (14)
PaO_2_ to FiO_2_ ratio >100 and <200	27 (68)	40 (80)
PaO_2_ to FiO_2_ ratio <100	7 (18)	3 (6)
**Infection severity at VAP onset**		
Uncomplicated infection	9 (23)	6 (12)
Sepsis	10 (25)	18 (36)
Septic shock	21 (53)	26 (52)
**Bacteraemic VAP**	13 (33)	16 (32)
**Augmented renal clearance**	8 (20)	7 (14)
**Acute kidney injury at VAP onset ^#^**	7 (19)	12 (33)
**De novo acute kidney injury ^§^**	13 (45)	17 (47)
**CRRT**	7 (18)	9 (18)
**vv-ECMO**	3 (8)	1 (2)
**Known respiratory CRAB colonization**	22 (55)	30 (60)
**Fast molecular diagnostics at VAP onset**	8 (20)	12 (24)
**Timely (<24 h) targeted therapy**	30 (75)	42 (84)
**Antibiotic therapy duration (days)**	13 (10–15)	12 (10–14)

Data are presented as no. (%) of included patients or as median (interquartile range), unless otherwise indicated. * *p* < 0.05 vs. cefiderocol group. ^#^ 6 IHD patients (4 patients in cefiderocol group, 2 patients in colistin group) were not included in analysis. ^§^ 6 IHD patients (4 patients in cefiderocol group, 2 patients in colistin group) and 19 patients with AKI at VAP onset (7 patients in cefiderocol group, 12 patients in colistin group) were not included in analysis. APACHE: Acute Physiologic Assessment and Chronic Health Evaluation; BMI: body max index; CRAB: carbapenem-resistant *Acinetobacter baumannii*; CRRT: continuous renal replacement therapy; SOFA: sequential organ failure assessment; VAP: ventilator-associated pneumonia; vv-ECMO: veno-venous extra-corporeal membrane oxygenation.

**Table 3:** Cox proportional hazard model for investigating predictors of clinical failure with first-line antimicrobial therapy.

	Univariable Analysis			Multivariable Analysis		
	aHR	95% CI	*p*-Value	aHR	95% CI	*p*-Value
Immunodepression	1.97	0.98–3.83	0.06	1.56	0.76–3.19	0.23
Charlson comorbidity index	1.28	1.12–1.47	<0.0001	1.21	1.04–1.42	0.01
SOFA score	1.15	1.02–1.30	0.02	1.07	0.92–1.25	0.35
Septic shock	1.91	0.93–3.87	0.07	1.52	0.69–3.33	0.29
Bacteremic VAP	1.46	0.74–2.90	0.28	/		
Augmented renal clearance	1.07	0.41–2.76	0.41	/		
CRRT	1.10	0.50–2.47	0.81	/		
Timely targeted therapy	0.44	0.22–0.90	0.02	0.40	0.19–0.84	0.01
Cefiderocol-based first-line regimens	0.37	0.17–0.79	0.01	0.38	0.17–0.85	0.02

CRRT: continuous renal replacement therapy; SOFA: sequential organ failure assessment; VAP: ventilator-associated pneumonia.

## Data Availability

The dataset can be made available by the corresponding author upon reasonable request.

## Supplementary Materials

The following supporting information can be downloaded at https://www.mdpi.com/article/10.3390/antibiotics12061048/s1. Table S1: IPTW-adjusted multivariable proportional Cox hazard model for investigating predictors of clinical failure; Table S2: Balance table before and after IPT weighting.

## References

[1] Vincent J.L., Sakr Y., Singer M., Martin-Loeches I., Machado F.R., Marshall J.C., Finfer S., Pelosi P., Brazzi L., Aditianingsih D. (2020). Prevalence and Outcomes of Infection Among Patients in Intensive Care Units in 2017. JAMA.

[2] Cai B., Echols R., Magee G., Arjona Ferreira J.C., Morgan G., Ariyasu M., Sawada T., Nagata T.D. (2017). Prevalence of Carbapenem-Resistant Gram-Negative Infections in the United States Predominated by *Acinetobacter baumannii* and *Pseudomonas aeruginosa*. Open Forum Infect. Dis..

[3] Segala F.V., Bavaro D.F., Di Gennaro F., Salvati F., Marotta C., Saracino A., Murri R., Fantoni M. (2021). Impact of SARS-CoV-2 Epidemic on Antimicrobial Resistance: A Literature Review. Viruses.

[4] Zilberberg M.D., Nathanson B.H., Sulham K., Fan W., Shorr A.F. (2016). Multidrug resistance, inappropriate empiric therapy, and hospital mortality in *Acinetobacter baumannii* pneumonia and sepsis. Crit. Care.

[5] Tacconelli E., Carrara E., Savoldi A., Harbarth S., Mendelson M., Monnet D.L., Pulcini C., Kahlmeter G., Kluytmans J., Carmeli Y. (2018). Discovery, research, and development of new antibiotics: The WHO priority list of antibiotic-resistant bacteria and tuberculosis. Lancet Infect. Dis..

[6] Paul M., Carrara E., Retamar P., Tängdén T., Bitterman R., Bonomo R.A., de Waele J., Daikos G.L., Akova M., Harbarth S. (2022). European Society of Clinical Microbiology and Infectious Diseases (ESCMID) guidelines for the treatment of infections caused by multidrug-resistant Gram-negative bacilli (endorsed by European society of intensive care medicine). Clin. Microbiol. Infect..

[7] Tamma P.D., Aitken S.L., Bonomo R.A., Mathers A.J., van Duin D., Clancy C.J. (2022). Infectious Diseases Society of America Guidance on the Treatment of AmpC β-Lactamase-Producing Enterobacterales, Carbapenem-Resistant *Acinetobacter baumannii*, and *Stenotrophomonas maltophilia* Infections. Clin. Infect. Dis..

[8] Pascale R., Pasquini Z., Bartoletti M., Caiazzo L., Fornaro G., Bussini L., Volpato F., Marchionni E., Rinaldi M., Trapani F. (2021). Cefiderocol treatment for carbapenem-resistant *Acinetobacter baumannii* infection in the ICU during the COVID-19 pandemic: A multicentre cohort study. JAC-Antimicrob. Resist..

[9] Falcone M., Tiseo G., Leonildi A., Della Sala L., Vecchione A., Barnini S., Farcomeni A., Menichetti F. (2022). Cefiderocol- Compared to Colistin-Based Regimens for the Treatment of Severe Infections Caused by Carbapenem-Resistant *Acinetobacter baumannii*. Antimicrob. Agents Chemother..

[10] Russo A., Bruni A., Gullì S., Borrazzo C., Quirino A., Lionello R., Serapide F., Garofalo E., Serraino R., Romeo F. (2023). Efficacy of cefiderocol- versus colistin-containing regimen for treatment of bacteremic ventilator-associated pneumonia caused by carbapenem-resistant *Acinetobacter baumannii* in COVID-19 patients. Int. J. Antimicrob. Agents.

[11] The European Committee on Antimicrobial Susceptibility Testing (2023). Breakpoint Tables for Interpretation of MICs and Zone Diameters. Version 13.0. http://www.eucast.org.

[12] Kalil A.C., Metersky M.L., Klompas M., Muscedere J., Sweeney D.A., Palmer L.B., Napolitano L.M., O’Grady N.P., Bartlett J.G., Carratalà J. (2016). Management of Adults with Hospital-acquired and Ventilator-associated Pneumonia: 2016 Clinical Practice Guidelines by the Infectious Diseases Society of America and the American Thoracic Society. Clin. Infect. Dis..

[13] CDC/NHSN Surveillance Definitions for Ventilator-Associated Events (VAE). https://www.cdc.gov/nhsn/pdfs/pscmanual/10-vae_final.pdf.

[14] Singer M., Deutschman C.S., Seymour C.W., Shankar-Hari M., Annane D., Bauer M., Bellomo R., Bernard G.R., Chiche J.D., Coopersmith C.M. (2016). The Third International Consensus Definitions for Sepsis and Septic Shock (Sepsis-3). JAMA.

[15] Kellum J.A., Lameire N., KDIGO AKI Guideline Work Group (2013). Diagnosis, evaluation, and management of acute kidney injury: A KDIGO summary (Part 1). Crit. Care.

[16] Bilbao-Meseguer I., Rodríguez-Gascón A., Barrasa H., Isla A., Solinís M.Á. (2018). Augmented Renal Clearance in Critically Ill Patients: A Systematic Review. Clin. Pharmacokinet..

[17] Evans L., Rhodes A., Alhazzani W., Antonelli M., Coopersmith C.M., French C., Machado F.R., Mcintyre L., Ostermann M., Prescott H.C. (2021). Surviving sepsis campaign: International guidelines for management of sepsis and septic shock 2021. Intensive Care Med..

[18] De Backer D., Cecconi M., Chew M.S., Hajjar L., Monnet X., Ospina-Tascón G.A., Ostermann M., Pinsky M.R., Vincent J.L. (2022). A plea for personalization of the hemodynamic management of septic shock. Crit. Care.

[19] Gatti M., Viaggi B., Rossolini G.M., Pea F., Viale P. (2022). An Evidence-Based Multidisciplinary Approach Focused on Creating Algorithms for Targeted Therapy of Infection-Related Ventilator-Associated Complications (IVACs) Caused by *Pseudomonas aeruginosa* and *Acinetobacter baumannii* in Critically Ill Adult Patients. Antibiotics.

[20] Tsuji B.T., Pogue J.M., Zavascki A.P., Paul M., Daikos G.L., Forrest A., Giacobbe D.R., Viscoli C., Giamarellou H., Karaiskos I. (2019). International Consensus Guidelines for the Optimal Use of the Polymyxins: Endorsed by the American College of Clinical Pharmacy (ACCP), European Society of Clinical Microbiology and Infectious Diseases (ESCMID), Infectious Diseases Society of America (IDSA), International Society for Anti-infective Pharmacology (ISAP), Society of Critical Care Medicine (SCCM), and Society of Infectious Diseases Pharmacists (SIDP). Pharmacotherapy.

[21] Dalfino L., Puntillo F., Ondok M.J., Mosca A., Monno R., Coppolecchia S., Spada M.L., Bruno F., Brienza N. (2015). Colistin-associated Acute Kidney Injury in Severely Ill Patients: A Step Toward a Better Renal Care? A Prospective Cohort Study. Clin. Infect. Dis..

[22] Fectroja, INN-Cefiderocol–European Medicine Agency. https://www.ema.europa.eu/en/documents/product-information/fetcroja-epar-product-information_en.pdf.

[23] Loeuille G., Vigneron J., D’Huart E., Charmillon A., Demoré B. (2023). Physicochemical stability of cefiderocol, a novel siderophore cephalosporin, in syringes at 62.5 mg/mL for continuous administration in intensive care units. Eur. J. Hosp. Pharm..

[24] Chawla L.S., Bellomo R., Bihorac A., Goldstein S.L., Siew E.D., Bagshaw S.M., Bittleman D., Cruz D., Endre Z., Fitzgerald R.L. (2017). Acute Disease Quality Initiative Workgroup 16.: Acute kidney disease and renal recovery: Consensus report of the acute disease quality initiative (ADQI) 16 workgroup. Nat. Rev. Nephrol..

[25] Ehrmann S., Luyt C.E. (2020). Optimizing aerosol delivery of antibiotics in ventilated patients. Curr. Opin. Infect. Dis..

[26] https://www.eucast.org/fileadmin/src/media/PDFs/EUCAST_files/Guidance_documents/Colistin_guidance_2022.pdf.

[27] https://www.eucast.org/fileadmin/src/media/PDFs/EUCAST_files/Rationale_documents/Cefiderocol_Rationale_Document_1.1_20220411.pdf.

[28] Amoah J., Stuart E.A., Cosgrove S.E., Harris A.D., Han J.H., Lautenbach E., Tamma P.D. (2020). Comparing Propensity Score Methods Versus Traditional Regression Analysis for the Evaluation of Observational Data: A Case Study Evaluating the Treatment of Gram-Negative Bloodstream Infections. Clin. Infect. Dis..

[29] Eikenboom A.M., Le Cessie S., Waernbaum I., Groenwold R.H.H., de Boer M.G.J. (2022). Quality of Conduct and Reporting of Propensity Score Methods in Studies Investigating the Effectiveness of Antimicrobial Therapy. Open Forum Infect. Dis..

[30] Peiffer-Smadja N., Bouadma L., Mathy V., Allouche K., Patrier J., Reboul M., Montravers P., Timsit J.F., Armand-Lefevre L. (2020). Performance and impact of a multiplex PCR in ICU patients with ventilator-associated pneumonia or ventilated hospital-acquired pneumonia. Crit. Care.

[31] Drwiega E.N., Rodvold K.A. (2022). Penetration of Antibacterial Agents into Pulmonary Epithelial Lining Fluid: An Update. Clin. Pharmacokinet..

[32] Eljaaly K., Bidell M.R., Gandhi R.G., Alshehri S., Enani M.A., Al-Jedai A., Lee T.C. (2021). Colistin Nephrotoxicity: Meta-Analysis of Randomized Controlled Trials. Open Forum Infect. Dis..

[33] Aydemir H., Akduman D., Piskin N., Comert F., Horuz E., Terzi A., Kokturk F., Ornek T., Celebi G. (2013). Colistin vs. the combination of colistin and rifampicin for the treatment of carbapenem-resistant *Acinetobacter baumannii* ventilator-associated pneumonia. Epidemiol. Infect..

[34] Candel F.J., Santerre Henriksen A., Longshaw C., Yamano Y., Oliver A. (2022). In vitro activity of the novel siderophore cephalosporin, cefiderocol, in Gram-negative pathogens in Europe by site of infection. Clin. Microbiol. Infect..

[35] Kawaguchi N., Katsube T., Echols R., Wajima T., Nicolau D.P. (2022). Intrapulmonary Pharmacokinetic Modeling and Simulation of Cefiderocol, a Parenteral Siderophore Cephalosporin, in Patients with Pneumonia and Healthy Subjects. J. Clin. Pharmacol..

[36] Katsube T., Nicolau D.P., Rodvold K.A., Wunderink R.G., Echols R., Matsunaga Y., Menon A., Portsmouth S., Wajima T. (2021). Intrapulmonary pharmacokinetic profile of cefiderocol in mechanically ventilated patients with pneumonia. J. Antimicrob. Chemother..

[37] Bassetti M., Echols R., Matsunaga Y., Ariyasu M., Doi Y., Ferrer R., Lodise T.P., Naas T., Niki Y., Paterson D.L. (2021). Efficacy and safety of cefiderocol or best available therapy for the treatment of serious infections caused by carbapenem-resistant Gram-negative bacteria (CREDIBLE-CR): A randomised, open-label, multicentre, pathogen-focused, descriptive, phase 3 trial. Lancet Infect. Dis..

[38] François B., Laterre P.F., Luyt C.E., Chastre J. (2020). The challenge of ventilator-associated pneumonia diagnosis in COVID-19 patients. Crit. Care.

[39] Karakonstantis S., Kritsotakis E.I. (2021). Systematic review and meta-analysis of the proportion and associated mortality of polymicrobial (vs monomicrobial) pulmonary and bloodstream infections by *Acinetobacter baumannii* complex. Infection.

[40] Alshaer M.H., Maranchick N., Bai C., Maguigan K.L., Shoulders B., Felton T.W., Mathew S.K., Mardini M.T., Peloquin C.A. (2022). Using Machine Learning To Define the Impact of Beta-Lactam Early and Cumulative Target Attainment on Outcomes in Intensive Care Unit Patients with Hospital-Acquired and Ventilator-Associated Pneumonia. Antimicrob. Agents Chemother..

[41] Xiao A.J., Miller B.W., Huntington J.A., Nicolau D.P. (2016). Ceftolozane/Tazobactam Pharmacokinetic/Pharmacodynamic-Derived Dose Justification for Phase 3 Studies in Patients With Nosocomial Pneumonia. J. Clin. Pharmacol..

[42] Tumbarello M., Raffaelli F., Giannella M., Mantengoli E., Mularoni A., Venditti M., De Rosa F.G., Sarmati L., Bassetti M., Brindicci G. (2021). Ceftazidime-Avibactam Use for Klebsiella pneumoniae Carbapenemase-Producing K. pneumoniae Infections: A Retrospective Observational Multicenter Study. Clin. Infect. Dis..

[43] Gatti M., Bartoletti M., Cojutti P.G., Gaibani P., Conti M., Giannella M., Viale P., Pea F. (2021). A descriptive case series of pharmacokinetic/pharmacodynamic target attainment and microbiological outcome in critically ill patients with documented severe extensively drug-resistant *Acinetobacter baumannii* bloodstream infection and/or ventilator-associated pneumonia treated with cefiderocol. J. Glob. Antimicrob. Resist..

[44] De Waele J.J., Lipman J., Akova M., Bassetti M., Dimopoulos G., Kaukonen M., Koulenti D., Martin C., Montravers P., Rello J. (2014). Risk factors for target non-attainment during empirical treatment with β-lactam antibiotics in critically ill patients. Intensive Care Med..

[45] Vardakas K.Z., Voulgaris G.L., Maliaros A., Samonis G., Falagas M.E. (2018). Prolonged versus short-term intravenous infusion of antipseudomonal β-lactams for patients with sepsis: A systematic review and meta-analysis of randomised trials. Lancet Infect. Dis..

[46] Crass R.L., Rodvold K.A., Mueller B.A., Pai M.P. (2019). Renal dosing of antibiotics: Are we jumping the gun?. Clin. Infect. Dis..

[47] McGovern P.C., Wible M., El-Tahtawy A., Biswas P., Meyer R.D. (2013). All-cause mortality imbalance in the tigecycline phase 3 and 4 clinical trials. Int. J. Antimicrob. Agents.

[48] Hoste E.A., Bagshaw S.M., Bellomo R., Cely C.M., Colman R., Cruz D.N., Edipidis K., Forni L.G., Gomersall C.D., Govil D. (2015). Epidemiology of acute kidney injury in critically ill patients: The multinational AKI-EPI study. Intensive Care Med..

[49] Brienza N., Giglio M.T., Dalfino L. (2012). Protocoled resuscitation and the prevention of acute kidney injury. Curr. Opin. Crit. Care.

