# Supplementary material for: Effectiveness of First-Line Therapy with Old and Novel Antibiotics in Ventilator-Associated Pneumonia Caused by Carbapenem-Resistant Acinetobacter baumannii: A Real Life, Prospective, Observational, Single-Center Study
Source: Antibiotics (Basel). 2023 Jun 14;12(6):1048. doi: 10.3390/antibiotics12061048 (PMC10295543; doi:10.3390/antibiotics12061048)
Supplement: Supplementary file 1 [file antibiotics-12-01048-s001.zip › antibiotics-2442195-supplementary.pdf]

Table S1. IPTW-adjusted multivariable proportional Cox hazard model to investigate predictors of clinical failure.

	aHR	95% CI	p-value
Severe immunosuppression	1.79	0.78 – 4.08	.2
CCI	1.18	1.01 – 1.36	.03
SOFA score	1.04	0.90 – 1.20	.56
Septic shock	1.47	0.67 – 3.22	.3
Bacteremic VAP	/		
ARC	/		
CRRT	/		
Appropriate treatment start within 24 hours from infection onset	0.32	0.16 – 0.68	.003
Cefiderocol-based regimens vs. colistin-based regimens	0.37	0.18 – 0.76	.007

CCI: Charlson comorbidity index; SOFA: sequential organ failure assessment; VAP: ventilator associated pneumonia; ARC: augmented renal clearance; CRRT: continuous renal replacement therapy.

Table S2. Balance table before and after IPT weighting

	Before IPTW			After IPTW		
	Cefiderocol group	Colistin group	SD	Cefiderocol group	Colistin group	SD
CCI, mean	5	5	20%	4	5	2%
SOFA, mean	10	9	10%	10	9	0.1%
Septic shock, %	53	52	1%	52	52	0.1%
Bacteremic VAP, %	33	32	1%	32	32	0.1%
ARC, %	20	14	16%	23	13	0.1%
CRRT, %	18	18	1%	16	18	0.1%
Severely immunocompromised, %	33	28	10%	35	27	0.1%
Antimicrobial start within 24 hours from infection onset, %	75	84	22%	69	85	0.3%

CCI: Charlson comorbidity index; SOFA: sequential organ failure assessment; VAP: ventilator associated pneumonia; ARC: augmented renal clearance; CRRT: continuous renal replacement therapy; IPTW: inverse probability treatment weighting; SD: standardized differences.
